# A Multidisciplinary View on Animal Welfare and Alternative Protein: Convergences and Perspectives from Professionals in Agricultural, Food, and Veterinary Sciences

**DOI:** 10.3390/foods14122140

**Published:** 2025-06-19

**Authors:** Iliani Patinho, Robson Mateus Freitas Silveira, Erick Saldaña, Alessandra Arno, Sérgio Luís de Castro Júnior, Iran José Oliveira da Silva

**Affiliations:** 1 Department of Food Science and Technology, Luiz de Queiroz College of Agriculture, University of Sao Paulo, Piracicaba 13418-900, SP, Brazil; 2 Enrivonment Livestock Research Center (Núcleo de Pesquisa em Ambiência—NUPEA), Department of Biosystems Engeneering, Luiz de Queiroz College of Agriculture, University of São Paulo (ESALQ/USP), Av. Pádua Dias, 11, Piracicaba 13418-900, SP, Brazil; alessandraarno123@gmail.com (A.A.); sergio.castro@usp.br (S.L.d.C.J.); iranoliveira@usp.br (I.J.O.d.S.); 3 Department of Animal Science, Luiz de Queiroz College of Agriculture, University of São Paulo (ESALQ/USP), Av. Pádua Dias, 11, Piracicaba 13418-900, SP, Brazil; 4 Sensory Analysis and Consumer Study Group, Escuela Profesional de Ingeniería Agroindustrial, Universidad Nacional de Moquegua, Prolongaci’ on Calle Ancash s/n, Moquegua 18001, Peru; erick_16_13@hotmail.com

**Keywords:** consumer behavior, animal-based products, cell-based meat, edible insects

## Abstract

This study investigated the perceptions of animal welfare and the consumption of alternative protein sources among future professionals in agronomy, food science, and veterinary medicine. A sample of 769 participants from three faculties [ESALQ (“Luiz de Queiroz” College of Agriculture), FZEA (School of Animal Science and Food Engineering), and FMVZ (School of Veterinary Medicine and Animal Science)] of the University of São Paulo was used. These faculties have different teaching focuses: agronomy, food and animal production, and veterinary, respectively. A relationship between the perception of animal welfare and alternative sources of protein based on the participants’ educational background was verified, specifically: (i) participants from the FZEA (food science) and FMVZ (veterinary) units would be interested in consuming farmed meat and expressed interest in trying it; (ii) students from the ESALQ (agronomy) have a low level of knowledge about animal welfare and are not very interested in knowing how animals are reared, and few participants attribute the presence of the health inspection seal as influencing their purchasing intention; (iii) participants, regardless of their academic background, did not express an intention to reduce their red meat consumption; (iv) the ESALQ was the campus which showed the most skepticism about animal sentience; (v) most participants from the FMVZ and FZEA reported being willing to pay 4–5% more for products that guarantee animal welfare. The findings suggest that the academic context influences individuals’ perceptions and food choices, highlighting the need for educational strategies that foster a greater awareness of animal welfare, encourage the adoption of more sustainable practices, and promote the acceptance of alternative protein sources within the agri-food sector.

## 1. Introduction

Our ancestors began preparing food millions of years ago, and throughout human evolution, many challenges related to food production and consumption have been faced [1]. Nowadays, the major challenge we face is how to intensify the production of animal source foods, considering that population growth is expected to reach 9.7 billion people in the coming decades. The FAO projects that global meat production is expected to increase by 41 million tons between 2020 and 2032, reaching a total of 382 million tons [2,3,4]. It should also be noted that the global trend of animal product consumption is not uniform, showing possible stability in North American and European countries and increased demand in Latin America, Africa, and Asia [5,6]. Thus, the large numbers surrounding the current consumption of animal products and their future prospects carry consequences for the environment, human health, and global food security [7].

In this scenario, the way animals are bred and slaughtered raises the interest of consumers, requiring the food industry to reconsider its production strategies [8,9,10]. As a result, consumer concerns about animal welfare during the production process and the sustainability of production systems have been increasing. These concerns are reflected in shifts in dietary habits, with an increasing number of people adopting more restrictive diets, such as vegetarianism and veganism [11,12,13], which drive the industry to create stamps and certifications which promote more sustainable animal products [14,15,16] and develop products using new alternative protein sources. Therefore, in the current discussions on the trends and changes in the consumption of animal source foods, three important concepts emerge: animal welfare, insect-based foods, and laboratory-grown meat.

Animal welfare is one of the main drivers behind investments in alternative production systems. Beyond being a scientific concern, it also encompasses ethical considerations, consumer perceptions, concerns, and beliefs, all of which can influence purchasing intentions regarding animal-based food products [17,18]. Subsequently, the greater demand for products which guarantee animal welfare requires the commitment of producers, clear government policies, the positioning of the food industry, and actions to be performed by other elements of the production chain [19]. Thus, the investigation of consumers’ perceptions on the welfare of farm animals has been widely studied in several studies [17,20,21] with divergent results between countries, genders, ages, religions, occupations, and educational and socio-economic levels, among others.

In addition to animal welfare concerns, conventional production systems are often regarded as unsustainable [5]. In this scenario, in an attempt to identify alternative and economically viable sources of protein production, the interest in research and investment in the use of insects in food preparations is increasing [22,23]. As food present in the human diet since antiquity and of great importance in Eastern culture, insects are sources of proteins, unsaturated fats, and other important bioactive substances such as carbohydrates, minerals, antioxidants, and vitamins [24]. In contrast, several questions arise about the consumption of insects, especially in Western society [25,26,27]. Consumer perception of insect food security represents the main barrier, since many cultures label them as “impure”, “pests”, and “transmitters of disease”, fostering the rejection of insect-based products.

The third consumption trend presented in this study concerns the production of cell-based meat, also known as artificial, in vitro, cultured, laboratory, clean, or synthetic meat [28,29]. Although there are some barriers to be trespassed, the advantages of cell-based meat production include reduced land use compared with conventional animal production, lower greenhouse gas emissions, and favorable dialogs on the ethical concerns involving animal welfare and the environmental impacts of livestock farming [5,30,31]. The acceptance of cell-based meat was researched under consumer perception and both favorable and contradictory results were reported [32,33]. For example, in the United States, nine states have so far implemented bans on lab-grown meat, reflecting growing regulatory resistance to this emerging technology [34].

In light of these trends, this study identifies a gap in the scientific literature. Although there is growing interest in research on the perception of final consumers regarding animal welfare and alternative protein sources, less attention is given to the perceptions of future professionals who will work in animal production chains and in the scientific community [33]. The university has, among its attributions, the characteristic of educating its students according to the future demands and trends expected for the most diverse professions. Thus, the knowledge and commitment of professors, students, and other members of the academic community about animal welfare and sustainable diets are crucial to shaping the next generation of professionals to work in the industry. Specifically in the Brazilian context, evaluating the attitudes and perceptions of students in the agricultural, food, and veterinary sciences—as well as the academic community’s engagement with the aforementioned concepts—is essential, as these individuals will soon be responsible for the production of animal source foods.

To this end, this study focused on three agricultural science units of the University of São Paulo. Within this context, the following research questions were posed: (a) What are the perceptions and attitudes of the academic community toward animal welfare? (b) What are their views on the consumption of animal products? (c) How does the academic community engage with the emerging trend of alternative protein sources?

## 2. Materials and Methods

### 2.1. Characterization of Selected Units

This study considers, as an academic community, a group of students (undergraduate and graduate), employees, and professors at the University of São Paulo (USP), Brazil. For this study, the units responsible for offering agricultural science courses were selected, using their approach to animal production as selection criteria. Table 1 presents the selected units and their respective approaches. In addition to undergraduate and graduate students, ESALQ has 184 professors and 432 technical–administrative staff. FZEA, in turn, has 106 professors and 129 technical–administrative staff. FMVZ, on the other hand, consists of 82 professors and 238 technical–administrative staff [35].

It is noted that the units manage different courses, resulting in varying numbers of undergraduate and graduate students. Additionally, ESALQ offers programs which are not directly related to animal production, such as Business Administration, Economics, and Forest Engineering. This difference may be associated with the specific focus of each campus, given that FZEA and FMVZ have a student profile with similar sociodemographic characteristics.

### 2.2. Participants

The strategy adopted was non-probabilistic convenience sampling, aiming to reach the largest possible number of respondents within the target audience of the research. The survey was disseminated through various channels, including institutional emails using the University of São Paulo’s databases, social media, university WhatsApp groups, and professional contacts, in order to maximize the reach and diversity of participants. There was no predefined limit for the number of responses, as the objective was to explore patterns of opinion, perception, or behavior with broad empirical representativeness, even without statistical inference to a defined population. Notices encouraging participation were sent periodically by e-mail and reminders were posted on social networks. The criteria for the selection of participants were: (a) being a regular consumer of animal products (for example, who consume animal products three or four times a week); (b) being an undergraduate or graduate student at the USP units selected in this study; (c) being an employee or professor at these same units; (d) presenting an interest and availability to participate in the study. The questionnaire was open for 10 months, obtaining a total of 769 valid answers.

### 2.3. Questionnaire

The questionnaire was developed in Compusense Cloud version 23.0.3 (Compusense Inc., Guelph, ON, Canada) and comprised three sections, covering a total of 21 questions adapted from previous works [20,33,36,37]. The sections were labeled as: *(1) Sociodemographic profile of respondents*; *(2) Dietary habits*, which investigated the consumption habits of meat products and the attitude of participants toward reducing meat consumption and including products made with new protein sources in their diets; *(3) Knowledge of production systems*, addressing the participants’ level of knowledge, respect, and attitudes toward animal welfare.

### 2.4. Data Analysis

Since all variables were categorical, associations between variables were assessed using the chi-square (χ^2^) test of independence.

The answers to questions 1 to 11 (Q1 to Q11) were handled by a Multiple Correspondence Analysis (MCA), excluding the other questions that had a different answer format (for these questions, the answers were analyzed using the frequency of answers). In MCA, the consumers are in rows and the questions in columns [38]. Within each cell there are only two possible answers: 1 (presence) and 0 (absence) [39]. The associations between questions are based on the chi-square distance between the different categories of variables given by each participant [33]. The analysis was performed using the R software (version 4.0.0) with the help of the ExPosition package (version 2.8.13) [40].

## 3. Results

### 3.1. Sociodemographic Profile

The sociodemographic profile of the participants, according to the USP training school, is presented in Table 2. The variables, gender, education level, age, education level, and family income, showed a statistically significant association with the analyzed campuses, as demonstrated by Pearson’s chi-square test (*p* < 0.001).

### 3.2. Responses to Questionnaire Questions

Table 3 shows the questions that were applied to evaluate the perceptions and attitudes of respondents, as well as the percentage obtained for each campus. These questions were selected on the assumption that the respondents would be free to express their point of view, since up to the time of the questions no additional information was provided to the respondents. The variables regarding food purchasing habits (Q1), substitution of animal source proteins (Q2), reduction in animal source product consumption (Q5), availability of information about animal welfare (Q8), and labeling of animal-based product conditions (Q10) did not show a statistically significant association between the campuses, as indicated by the chi-square test results (*p*-values: 0.104, 0.316, 0.557, 0.155, and 0.922, respectively). On the other hand, the other variables demonstrated a significant association (*p* < 0.05). To better clarify the relationship between these variables and the three campuses, we conducted a correspondence analysis.

### 3.3. Multivariate Representation of Campuses Based on the Questionnaire

**Figure 1:**
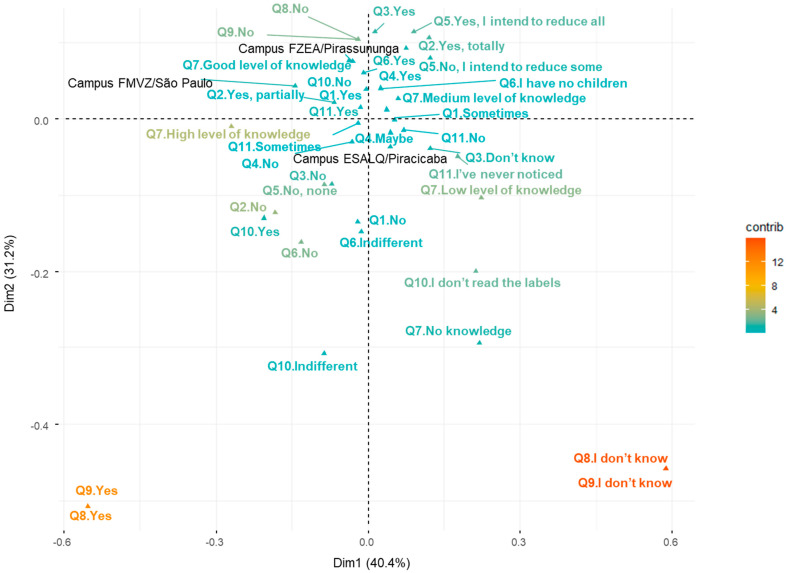
Multiple Correspondence Analysis (MCA) performed with Q1 to Q11 questions, at the three USP campuses. The details of the questions are shown in Table 3.

Figure 1 presents the results of the MCA, based on the responses to questions Q1 to Q11 across the three campuses of the USP. In the plot, each point represents a specific response level to one of the questions, positioned in a two-dimensional Euclidean space. It allows the visualization of association patterns among the categorical variables in the study.

The two main dimensions extracted by the MCA jointly explain 71.6% of the total variance in the data [Dimension 1 (Dim1; 40.4%) and Dimension 2 (Dim2; 31.2%)]. These dimensions summarize the main axes of variation in the responses, capturing the most relevant contrasts among the campuses. In general, the closer a point is to the center, the lower its contribution to the overall structure; points located on the periphery have a greater influence on defining associations between responses.

The main separation between groups occurs along Dim 2. Participants from the FZEA are concentrated in the upper region, relatively close to those from the FMVZ, while those from the ESALQ are predominantly located in the lower region of the graph. This spatial distribution suggests notable differences among the campuses regarding the perceptions and attitudes toward animal welfare, the consumption of animal products, and knowledge of these topics.

Different response patterns can be interpreted from the analysis. For instance, in Q1 (being responsible for household food purchases), participants who answered “Yes” are more associated with the ESALQ and FZEA campuses. In Q2, the answer “No” to the possibility of replacing animal protein with other sources is clearly associated with the ESALQ participants. In Q5, individuals from the FZEA and FMVZ expressed a stronger intention to partially or totally reduce the consumption of animal products, especially red meat, while those from the ESALQ mostly answered “No” to this intention.

Regarding knowledge about farm animal welfare (Q7), participants from the FZEA and FMVZ demonstrate good to medium levels of knowledge, while those from the ESALQ exhibit more heterogeneity, encompassing both high and low levels of awareness. For Q10, the response “No” regarding whether labels allow the identification of animal breeding conditions was common across all campuses. In Q11, when asked whether the presence of a health inspection seal influences purchasing decisions, FMVZ and FZEA participants tended to respond “Yes,” while ESALQ participants showed more variation, responding “No”, “Sometimes”, or “Never noticed.”

The MCA also highlighted important groupings around questions Q8 and Q9. Participants who answered “Don’t know” formed a cohesive group, representing a profile of lack of opinion or knowledge about the availability of information in Brazil on animal welfare and breeding conditions. Conversely, those who answered “No” indicated a clear perception that such information is not available. Similarly, for Q3, participants who answered “No” are against consuming lab-grown meat, while those who answered “Yes” are in favor. The “Don’t know” response again indicates unfamiliarity with the topic.

Finally, for Q4 (consumption of insect-based food products), participants who responded “Yes” expressed openness to alternative protein sources, while a high proportion of “Maybe” responses—especially from the FZEA and ESALQ—reflects indecision.

Thus, Figure 1 provides an integrated view of the response patterns across campuses, revealing three main participant profiles: those who are more favorable to conscious consumption and knowledgeable about the topic (FZEA and FMVZ), those showing greater heterogeneity or lack of awareness (ESALQ), and a third group distinguished by neutral or uninformed responses (visible in the extreme quadrants of the figure). The clarity of this graphical representation, supported by the interpretation of the dimensions, contributes to an understanding of participants’ attitudes and perceptions toward animal welfare and the consumption of animal-derived products.

### 3.4. Consumption Habits

**Figure 2:**
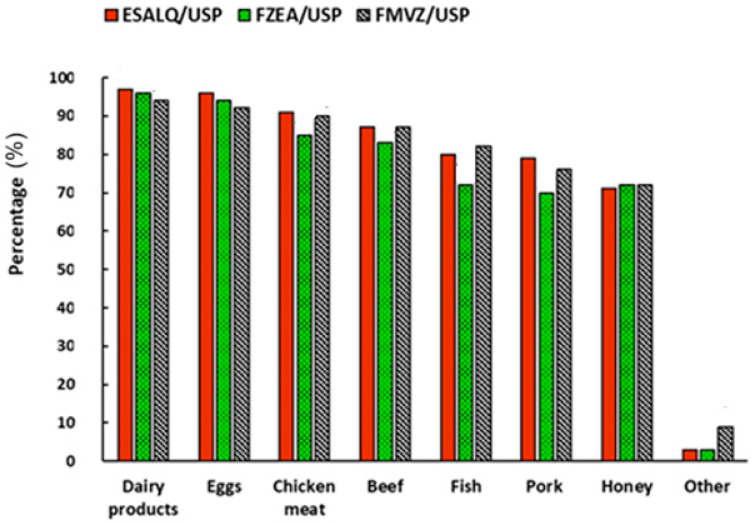
Consumption habits of animal products from participants’ responses.

Another important issue refers to the attitudes of the participants regarding consumption habits, as can be verified in Figure 2. It can be observed that participants from all campuses demonstrated similar behavior in consumption habits. Considering all the participants in the study, the general consumption of the USP’s agricultural sciences academic community was 95.6% dairy products, 94% for eggs, 88.6% chicken meat, 85.5% beef, 78% fish, 75% pork, 71.6% honey, and 5% other (e.g., sheep meat, rabbits, and seafood). It should be noted that the FZEA presented the lowest meat consumption among the USP units and the FMVZ showed a greater interest in the consumption of non-conventional animal products (others).

**Figure 3:**
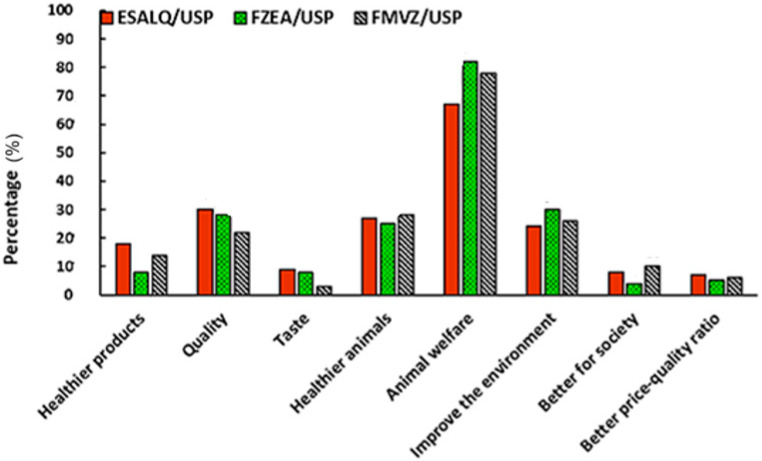
Most important reasons for purchasing animal products that respect animal welfare in the participants’ opinions.

When asked what would be the main reasons for purchasing products that respect animal welfare, participants expressed their opinion (Figure 3). Considering the participation of all respondents, 75.6% attributed welfare as the first reason. This greater attribution to animal welfare is expected, because animal welfare certification, although directly and indirectly associated with other quality attributes of animal source products, does not necessarily guarantee that these are met. The secondary reasons were improvements to the environment, healthier animals, and quality, all with 26.6%. Comparing the units, the FZEA, which had the lowest meat consumption, attributed the highest percentage to animal welfare (82%), followed by the FMVZ (78%) and ESALQ (67%).

**Figure 4:**
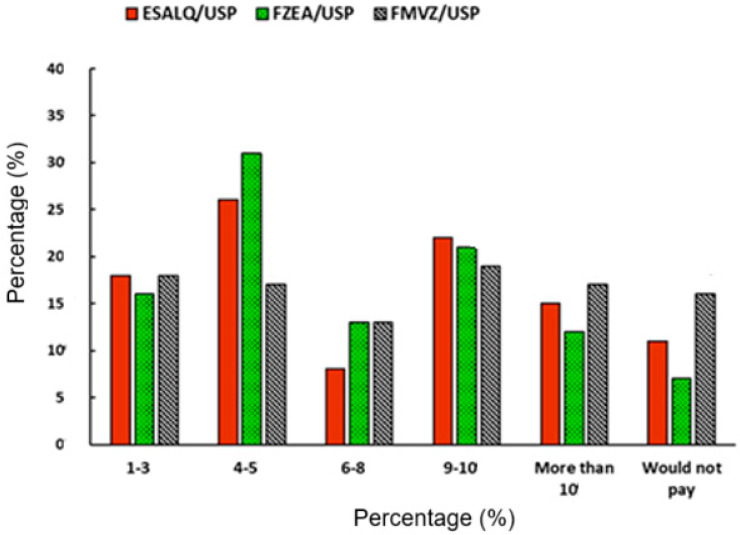
Percentage of participants that would pay for products that ensure animal welfare.

Figure 4 shows the percentage of participants willing to pay higher prices for products that guarantee animal welfare. Both the ESALQ (26% of respondents) and FZEA (31%) participants showed a greater interest in paying 4–5% more for certified products. On the other hand, the FMVZ academic community was the most reluctant to overpay for such products, with 16% of its respondents unwilling to pay more (compared with 11% from the ESALQ and 7% from FZEA). The response ‘would not pay’ may represent two opposing groups: individuals who believe it is not worth the investment to acquire products that guarantee animal welfare and those who would not overpay because they believe welfare should be a basic requirement for every animal product.

**Figure 5:**
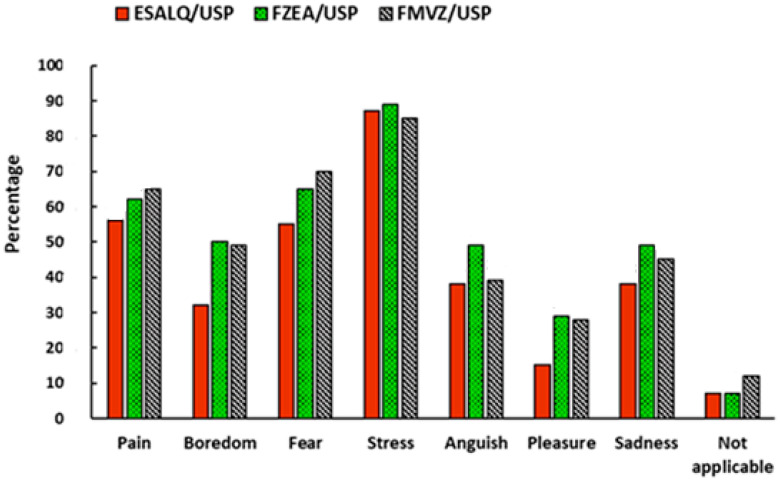
Animal sensory approach: participants’ opinions on what animals feel.

In order to obtain a pertinent answer about the participants’ perception of the sentience of animals, we asked: can animals reared for consumption, such as chickens, cattle and pigs, feel pain, fear, among others? The comparative results between the three units are presented in Figure 5. Considering all the research participants, 87% believe that the animals feel stress, followed by fear (63.6%), pain (61%), boredom (43.6%), sadness (44%), anguish (42%), and pleasure (24%). It is observed that the positive feeling of pleasure was the one that presented the lowest percentage of responses, which suggests that the participants tend to attribute a greater burden to the negative feelings of the animals. In addition, 8.66% of the total interviewees pointed out that none of the feelings apply to production animals. Comparing the units, the ESALQ presented the lowest percentages of feelings, except for stress. On the other hand, the FZEA presented the highest percentages for five of the seven feelings evaluated. Meanwhile, the scientific community at the FMVZ showed more sensitivity to feelings of pain and fear.

**Figure 6:**
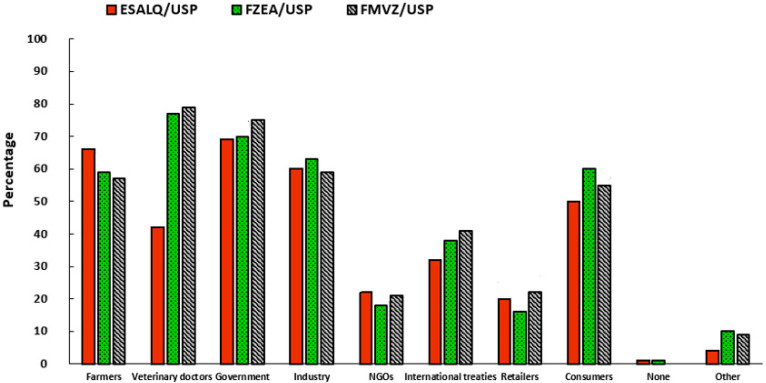
Responsibility to ensure animal welfare.

For welfare to be truly guaranteed in production systems, joint actions by scientists, farmers, governments, industries, retailers, welfare organizations, and consumers are needed. According to Figure 6, it is possible to observe how the participants assign responsibilities to these actors in the production chain. In general, considering all the interviewees, 71.3% believe that the responsibility lies with the government, followed by veterinary doctors (66%), industry and farmers (both with 60.6%), consumers (55%), international treaties (37%), NGOs (20.3%), and retailers (19.3%). In comparison, the FMVZ and FZEA attribute greater responsibility to veterinary doctors (79% and 77%, respectively), against 42% from ESALQ. The first two units offer a course in veterinary medicine, which suggests that there is a sense of self-responsibility in these scientific communities, which believe that they should actively act to ensure a higher level of animal welfare. The ESALQ, which does not offer a veterinary course, has assigned greater responsibilities to the government (69%) than other elements in the production chain. Although not among the main factors, all communities attribute considerable responsibility to consumers (60% from FZEA, 55% from FMVZ, and 50% from ESALQ).

## 4. Discussion

### 4.1. Perceptions and Attitudes of the Academic Community Regarding Animal Welfare

Concerns about animal welfare during pre-slaughter handling began to take shape in Europe over the centuries, influencing the discussions and practices in various parts of the world. In Brazil, for several decades, legislation has required attention to the welfare of animals destined for slaughter, establishing penalties for non-compliance. Over time, these regulations have been updated to ensure greater alignment with the ethical and technical standards demanded by society and international markets. Among the main regulatory milestones is Instruction No. 113, dated 16 December 2020, issued by the Ministry of Agriculture, Livestock and Supply (MAPA), which sets procedures to ensure animal welfare during the transport and pre-slaughter stages of pigs. Complementing this guideline, Ordinance N^o^. 365, dated 16 July 2021, regulates pre-slaughter handling and humane slaughter, establishing mandatory criteria for all stages—from fasting to stunning and slaughter—with a focus on reducing animal suffering. These regulations are aligned with the international model of the Five Domains of Animal Welfare, which considers not only physical aspects such as nutrition, environment, health, and behavior, but also the mental domain, recognizing the affective states of animals [41]. Thus, animal welfare results from the balanced interaction among animals, facilities, and people, making it essential to provide ongoing training for professionals and to adapt physical infrastructure to ensure ethical and responsible practices throughout the entire production chain.

From this context, in the present study, most participants from the FMVZ and FZEA reported being willing to pay 4–5% more for products that ensure animal welfare (Figure 4). One justification for this result may be related to the target audience of this study: which consisted primarily of students from veterinary and food courses who are attentive to animal welfare issues. However, it should be considered that the fact that participants admit they would pay more for products ensuring animal welfare may be an immediate response to the question and does not always directly reflect action at the time of purchase. This is confirmed in a study by the NGO, World Animal Protection, conducted with 1200 respondents in 72 municipalities in Brazil. In this study, about 70% of respondents considered that products with a production seal ensuring animal welfare are more expensive than products without this certification. Among those who intended to buy certified products, over 70% stated they would do so if the price were equal to that of non-certified alternatives [42]. However, it is important to note that a majority of respondents also reported not having access to animal welfare information at the point of purchase, which may limit their actual ability to identify or choose such products. Similarly, Gross et al. [43] indicated that German consumers are willing to pay more for organic ham and, to a lesser extent, for ham produced under different animal welfare standards.

Indeed, price is a key element in the purchasing decision and is used as an indicator of quality when there is insufficient information to assess the product and in situations of risk. According to Merlino et al. [44], purchasing products in Italy at a particularly high price represents protection against low-quality products.

Based on the results found in the present study, considering the importance of animal welfare for the Brazilians who participated in this research, the production chain, including farmers, food industries, and retailers, should consider a certification system to ensure the origin of the product and, at the same time, add value to the product and respect animal welfare. The results corroborate Miranda-de la Lama et al. [37], who indicated that some studies in Spain showed that consumers take animal welfare into account when purchasing products. However, these authors observed that retailers, based on their practical experience, perceived a low willingness of consumers to pay more for products that ensure animal welfare, with the main motivation for purchasing these products being related to perceived sensory quality, while concerns about animal welfare occupy a secondary role in purchasing decisions.

In general, in most studies where animal welfare is evaluated as a factor influencing the final quality of animal products, it is usually found that consumers view it as a positive attribute [45,46], meaning that the information is not directly available during purchase and consumption, but plays a fundamental role in the perception of quality [47].

Following the same line of research, Ennes [48] analyzed the profile of Brazilian meat consumers, focusing on their perception of aspects such as environmental impact, animal welfare, quality, and health. The study involved 1039 consumers from various regions of Brazil and indicated that gender plays a significant role in the choice of attributes valued at the time of purchase. Among women, aspects such as sustainability certifications and animal welfare were considered more relevant (*p* ≤ 0.05), highlighting the importance given to these factors in the product purchasing process. Another study conducted with consumers of animal-based products in Fortaleza, Ceará, Brazil, on the welfare of production animals, revealed that although most consumers did not have sufficient knowledge on the topic, they were willing to pay more for products that consider animal welfare. This willingness is linked to the belief that differentiated farming practices can improve the quality of the final product. Furthermore, consumers showed an interest in products with certification that attests to the quality and animal welfare practices [49].

In a global context, Mayfield et al. [50] evaluated whether consumers in Great Britain, Italy, and Sweden expected animal welfare to affect other qualities of the product. The researchers demonstrated that consumers associated animal welfare with positive influences on taste and health, interpreting these attributes as indicators of higher product quality and healthier animals.

Regarding animal sentience (Figure 5), it is worth noting that the majority of interviewees from all campuses believe that animals have feelings. This is important because all the institutions evaluated have facilities and practices that place the scientific community in direct contact with animal production. In turn, stress was the most evident element of suffering, receiving the highest percentage of responses. One possible justification is that stress is easier to observe and measure compared with other feelings, such as fear, pain, distress, pleasure, and sadness, which are more abstract concepts and difficult to observe directly [18].

The academic community that showed the most skepticism about animal sentience was the ESALQ. One point to consider is that the ESALQ is the only unit that does not have an undergraduate course specifically focused exclusively on animal production (such as zootechnics and veterinary medicine). Additionally, respondents from courses outside the agricultural sciences who are part of the ESALQ may have influenced the result. On the other hand, the FMVZ, a scientific community mainly composed of veterinary medicine students and faculty, showed the highest percentage of “not applicable” responses regarding animal feelings (Figure 5). Although contrary to the expectations of this research, this result aligns with the findings of Paul and Podberscek [51], who compared veterinary medicine students in England across different years of their degree. The authors found that, over the years, final-year students classified animals as having lower sensitivity levels compared with students just starting their degree.

Regarding the attribution of responsibility for animal welfare, the results of this study (Figure 6) corroborate the findings of Thorslund et al. [36]. The academic community evaluated in this article indicates that veterinarians, farmers, the government, the industry, and consumers are the main agents responsible for animal welfare. Academic communities that have veterinarians in their scientific staff assigned greater responsibility to themselves, demonstrating that these professionals and students recognize their importance in ensuring animal welfare in the production process. On the other hand, NGOs are not strongly considered responsible for animal welfare by the Brazilian scientific community, despite their active role in consumer awareness. The results align with a study conducted in Denmark and Norway, where Boogaard et al. [52] indicated that consumers assigned significant responsibility to farmers and themselves. When respondents point to responsibility for retailers, this also suggests an indirect responsibility of consumers themselves, as they are the ones who purchase these products [53]. Additionally, when respondents say that the government should increase the price of meat [54], they are expressing the opinion that the government should intervene in the meat market and support alternative or premium products, which could also be interpreted as a recognition of their own responsibility, as they would be willing to buy these products if the government acted this way.

Finally, it is important to highlight that the differences between the reality of the Brazilian scientific community in the Southeast Region of Brazil and those of other countries should be evaluated with caution, considering cultural, economic, and social aspects. It is known that the perception of food value can vary among consumers from different countries. The Brasil Food Trends 2020 report indicated that Brazil strongly adheres to the behavioral consumption trends observed in Europe, North America, and Asia. Approximately 21% of Brazilian consumers make food decisions based on aspects such as health, welfare, sustainability, and ethics, provided that they are backed by quality seals and information about the product’s origin [55]. This fact highlights the importance of different perceptions in isolated analyses and, at the same time, encompasses a global discussion on these issues.

### 4.2. Position of the Academic Community Regarding the Consumption of Animal Products

The consumption habits of animal-based products are influenced by various factors, including consumers’ personal characteristics and product properties. Based on the results of the present study, the academic community representing the three campuses of agronomy, food science, and veterinary science shows an interest in animal welfare but is not willing to reduce its consumption of animal-based products (Table 3), specifically red meat.

In the current scenario, many consumers, especially those with high incomes, wish to continue consuming meat while not wanting to contribute to animal suffering [31]. According to Hartmann & Siegrist [26], changing consumer eating behavior will not be easy, as it is linked to taste preferences, social norms, and culinary traditions. Furthermore, meat is highly valued by consumers for its nutritional value, taste, and other sensory characteristics [56], being perceived as a nutritious and healthy food that is part of a balanced diet. These findings challenge the study by González et al. [57], which reported a sharp decline in red meat consumption over the past decades in several countries worldwide. Although such a trend may reflect regional changes or specific consumer groups, data from the FAO [4] shows that global per capita meat consumption increased from 24.2 in 1964/66 to 36.4 kg in 1997/99, with projections indicating a rise to 45.3 kg by 2030. This growth is driven primarily by developing countries, such as China and Brazil, where per capita consumption increased from 11.4 to 25.5 kg during the same period.

Another relevant point to mention is that some studies suggest that consumers are unaware that meat production and consumption have a significant environmental impact. This observation applies to consumers in several Western countries, including the U.S., Germany, the Netherlands, Portugal, and Australia. Based on the results of four studies, only 18 and 38% of participants agreed with statements regarding the negative impact of meat consumption and production on the environment [26].

Gender differences were also investigated in three studies. One study found no effect [58], while two studies found that women perceive meat consumption as having a greater environmental impact compared with men [59]. This suggests that women are more likely to reduce meat consumption than men. Overall, various scientific findings suggest that only a minority of participants are willing to reduce meat consumption for ecological reasons [26]. These findings are similar to those of the present study.

When evaluating the consumption habits of respondents representing the academic community (Figure 2), no major discrepancies were observed among the evaluated units. The results align with findings from World Animal Protection [42], which indicate that chicken, eggs, and beef are the most consumed animal-based products.

Furthermore, in the present study, among different types of meat, pork is the least consumed by Brazilians. These findings corroborate data from other Latin American countries, where pork is among the products that Mexicans, Chileans, and Colombians report never purchasing [42].

In turn, the recent study by Ennes [48] revealed that chicken is the most consumed meat in Brazil, with 98.6% of participants reporting its consumption, followed by beef (98.3%) and fish (97.8%). Additionally, the consumption of different types of meat varies by gender and region of the country. Men tend to consume more buffalo, goat, sheep, pork, and organic meat, whereas women show a greater preference for meat alternatives. Regarding regional differences, buffalo meat is more consumed in the north, goat meat is predominant in the northeast and center–west, and sheep and pork are more popular in the south.

### 4.3. Dialog of the Academic Community with the Consumption Trends of Alternative Protein Sources

The possibility of food shortages, threats to human health, and environmental challenges—fueled by the growth and aging of the global population—are driving the pursuit of alternative and sustainable food sources [60]. In this context, consumers around the world are becoming increasingly concerned and are seeking to better understand how food is produced and marketed. In the present study, regarding the production of lab-grown meat, a significant percentage of participants from the FZEA (food science) and FMVZ (veterinary) units would be willing to consume this type of protein and expressed interest in trying it (Table 3). In this scenario, cultivated meat production has been gaining global prominence as a viable alternative for obtaining animal-based foods without the need to rear or capture animals. Currently, 156 companies in 26 countries are developing cultivated meat, producing beef, chicken, pork, and fish proteins through cell culture in laboratories [61].

In Brazil, research on alternative proteins has been expanding. Institutions such as Embrapa, in partnership with universities, have been dedicated to studying cultivated meat. However, Embrapa recently conducted a study on the consumer perception of cultivated meat after the COVID-19 pandemic. The results showed that many consumers are willing to try cultivated meat, but concerns remain regarding its safety, nutritional value, taste, texture, price, and production method. Furthermore, it is important to highlight that Brazil does not yet have specific legislation to regulate the production and commercialization of this meat [61].

Finally, it was observed that the profile of the potential cultivated meat consumer includes young participants (under 30 years old) with a high level of education and information. On the other hand, when considering future consumers, this younger generation will influence both purchasing power and the development of the next generation’s eating habits. Indeed, further research is recommended to assess consumer perceptions and expectations, especially in countries where culture or religion might hinder the acceptance of cultivated meat. To the best of our knowledge, cultivated meat production still faces significant challenges related to cost, scalability, and, most notably, sensory attributes, before it can reach the market and gain consumer acceptance. In the present study on foods produced from insects, the academic community of the ESALQ (agronomy) was more willing to try these products and differed statistically from the FZEA (food science) and FMVZ (veterinary medicine), which showed lower percentages. In the case of the FZEA, the values increased among those who answered “maybe” (Table 3), indicating that this type of product may attract the interest of these participants and possibly spark a desire for this sensory experience.

In Brazil, Lucchese-Cheung et al. [62] demonstrated that the taste of a biscuit made with larvae flour was positively evaluated and generated a greater willingness to try the product. However, the study by Soares Araújo et al. [63] showed that the main insects farmed in captivity in Brazil are intended solely for animal feed. According to Bisconsin-Júnior et al. [22], consumers from different regions of the country have distinct perceptions of edible insects, with culture being a determining factor. Clearly, disgust toward insects is a cultural construct, socialized among all members of a social group. Even farmers, who have greater exposure to insects, tend to approach them with fear and aversion [64].

Comparing different cultures, it was observed that in Nigeria, where insect consumption is common, 60% of surveyed higher education students consume insects, and 47.24% agree that governments should encourage this practice [65]. In contrast, in Iran, where insect consumption is not a common practice, only 23% of university students were in favor of the idea, and 15% moderately supported it [66]. In this context, in some countries, such as Belgium, the Food Safety Authority (FAVV-AFSCA) authorized the production and commercialization of ten insect species for human consumption in 2013 [56]. In Germany, insect-based foods have been approved for sale, and many supermarkets offer insect-based foods in the form of burgers, pasta, or protein bars [67], which aligns with the findings of Hartmann & Siegrist [26], who reported that people are more willing to consume processed insect products compared with unprocessed (fresh) insects.

However, the findings of these studies support the idea that insects should be promoted as an alternative to nuts, given that their texture, macronutrient content, and even flavor are already comparable. In fact, insects are already used in a similar way to nuts, as optional ingredients in some dishes, for example, coated in chocolate or mixed into baked goods, fried, roasted, or completely ground [68].

Another concern among researchers is the extinction of insects. In Hidalgo, Mexico, fourteen of the thirty insect species used as food are threatened due to commercialization [69]. In China, the Polyrhachis ant is endangered due to its use in brandy [70]. It is important to consider what would happen if cicadas, which are difficult to cultivate, became popular among Americans [68]. Therefore, breeding methods and regulations are essential to ensure sustainable practices.

Finally, the willingness to try alternative protein sources, such as cultured meat and insect-based foods, appears to be higher in scientific communities than in the general public [65], as dietary patterns are deeply rooted in culture and difficult to change. Furthermore, these protein sources are still expensive. The academic community, however, due to its greater exposure to innovations and market trends, seems to overcome barriers related to a lack of knowledge, potential biases, and taboos surrounding these new trends and disruptive technologies. In this context, van Huis [71] suggests that adequate information on insect sustainability, food safety, nutritional value, increased familiarity (experimental tasting), regulation, commercial availability, and media coverage are factors likely to drive consumer acceptance in the future.

## 5. Limitation of the Study

Although this study yielded relevant findings, several limitations should be acknowledged. The primary limitation concerns the gender imbalance observed in the FZEA and FMVZ campuses, which may have influenced other sociodemographic characteristics. Additionally, the use of an online questionnaire may have introduced self-selection bias, favoring the participation of students and researchers more engaged with the topic. Nevertheless, the results remain valid, given that the participants are or will be directly involved in animal production and the consumption (or avoidance) of alternative protein sources. Further research with larger and more diverse samples is essential to support and expand these findings, enabling a more representative understanding of consumer perceptions and choices regarding animal welfare and alternative proteins.

## 6. Conclusions and Future Perspectives

Respondents from Agricultural Sciences courses have little knowledge of animal welfare, unlike those in Food and Veterinary Sciences. In general, university participants demonstrate an interest in animal welfare and concern about the environmental impacts of conventional production; however, they do not demonstrate a willingness to reduce the consumption of these products. Furthermore, this study demonstrated that labels containing information about the conditions of rearing animals can encourage and promote the purchase of products of animal origin, adding value to them. Therefore, research institutions, regulatory agencies, and the private sector in Brazil must work together to promote animal welfare practices throughout the meat production chain in order to gain consumer trust.

Regarding alternative sources of protein, a positive percentage of the university community expressed a willingness to experiment with and, in general, consume laboratory-grown meat. Specifically, the academic community in agricultural sciences in Brazil has a significant responsibility, as it operates within one of the largest meat and animal products industries in the world. For this reason, understanding how the scientific community engages with and commits to these trends is of great importance. Thus, further studies are strongly encouraged to explore the role of higher education and scientific engagement in shaping more sustainable and welfare-conscious food systems.

## Figures and Tables

**Table 1:** Units selected from the University of São Paulo (USP).

Campus	City	Undergraduate Courses	Undergraduate Students	Graduate Students
“Luiz de Queiroz” College of Agriculture (**ESALQ**)	Piracicaba	Administration, Biology, Food Science, Economics, Agronomy, Forestry, Environmental Management, and License in Agricultural Science	2031	1262
College of Animal Science and Food Engineering (**FZEA**)	Pirassununga	Food Science, Biosystems Engineering, Veterinary Medicine, and Animal Science	1305	405
School of Veterinary Medicine and Animal Science (**FMVZ**)	São Paulo	Veterinary Medicine	726	666

**Table 2:** Sociodemographic profile of participants from different campuses (n = 769).

	ESALQ n = 486	FZEA n = 174	FMVZ n = 109
Gender (*p* < 0.001)			
Men	251	146	81
Women	235	28	28
Age (*p* < 0.001)			
<18	2	5	1
18–24	168	134	25
25–30	110	29	35
31–40	67	6	19
41–50	53	0	15
51–60	63	0	11
>60	23	0	3
Education Level (*p* < 0.001)			
High school	20	15	3
Incomplete higher education	163	122	24
Complete higher education	71	19	11
Incomplete postgraduate	79	12	38
Postgraduate (master’s or doctorate)	153	6	33
Family income (*p* < 0.001)			
Up to 3	227	144	52
4 to 7	126	37	27
8 to 10	52	9	6
11 to 15	52	8	18
Over 15	29	6	6

Note: based on the Brazilian minimum wage (BRL).

**Table 3:** Active variables, different response levels, and percentages obtained from each USP campus on respondents’ perceptions and attitudes.

Active Variables	ESALQ (486)	FZEA (174)	FMVZ (109)
Response Levels			
Q1. Do you buy food for your house? (*p* = 0.104)			
Yes	344	124	64
No	35	10	139
Sometimes	107	40	32
Q2. Would you substitute animal source proteins with other protein sources? (*p* = 0.316)			
Yes, totally	111	43	18
Yes, partially	262	85	58
No	113	46	33
Q3. Would you consume other types of proteins (ex: meat produced in a lab)? (*p* < 0.001)			
Yes	153	89	49
No	230	51	40
Don’t know	103	34	20
Q4. Would you consume insect-based foods? (*p* = 0.021)			
Yes	151	36	31
No	176	68	50
Maybe	159	70	28
Q5. Would you reduce your consumption of animal source products? (*p* = 0.557)			
Yes, I intend to reduce all	138	57	30
Yes, I intend to reduce some	87	25	15
No, none	261	92	64
Q6. Would you interfere in the quantity of food consumed by your children? (*p* < 0.001)			
Yes	124	30	20
No	126	16	24
Indifferent	27	5	4
I have no children	209	123	61
Q7. Level of knowledge about the conditions of the lives of livestock (*p* < 0.001)			
No knowledge	17	2	4
Low level of knowledge	104	18	11
Medium level of knowledge	195	47	17
Good level of knowledge	100	65	31
High level of knowledge	70	42	46
Q8. Is there sufficient information in your country about the well-being of animals? (*p* = 0.155)			
Yes	37	14	9
No	390	146	95
I don’t know	59	14	5
Q9. Would you like to be informed on the conditions of animal rearing? (*p* = 0.007)			
Yes	399	161	91
No	45	6	5
I don’t know	42	4	4
Q10. Do the packages of animal-based products allow for identifying the conditions the animals were reared in? (*p* = 0.922)			
Yes	34	11	7
No	398	143	94
I don’t read the labels	44	17	6
Indifferent	10	3	2
Q11. Does the presence of the sanitary inspection label influence your intention to purchase? (*p* < 0.001)			
Yes	243	112	74
No	39	3	4
Sometimes	97	27	11
I’ve never noticed	107	32	20

## Data Availability

The original contributions presented in the study are included in the article, further inquiries can be directed to the corresponding authors.
